# Imaging findings and outcomes in patients with carotid cavernous fistula at Inkosi Albert Luthuli Central Hospital in Durban

**DOI:** 10.4102/sajr.v22i1.1264

**Published:** 2018-01-25

**Authors:** Nasr Timol, Khatija Amod, Rohen Harrichandparsad, Royston Duncan, Tarylee Reddy

**Affiliations:** 1 Department of Radiology, University of KwaZulu-Natal, South Africa; 2 Department of Health, University of KwaZulu-Natal, South Africa; 3 Department of Radiology, Inkosi Albert Luthuli Central Hospital, South Africa; 4 Department of Neurosurgery, University of KwaZulu-Natal and Inkosi Albert Luthuli Central Hospital, South Africa; 5 Department of Radiology, Lake Smith & Partners and University of KwaZulu-Natal, South Africa; 6 Biostatistics Unit, Medical Research Council, South Africa

## Abstract

**Background:**

Carotid cavernous fistulas (CCFs) are relatively uncommon and are difficult to diagnose clinically. Radiological imaging plays a significant role in making the diagnosis with recent advances improving the ability of radiologists to diagnose the condition. Despite these developments, digital subtracted angiography (DSA) remains the gold standard in diagnosing CCFs and simultaneously provides the opportunity for intervention.

**Objectives:**

To determine the imaging findings of patients presenting to Inkosi Albert Luthuli Central Hospital (IALCH) with a CCF and to assess the outcome of endovascular intervention.

**Method:**

We reviewed the electronic records and archived imaging data of consecutive patients diagnosed with CCF between January 2003 and May 2016 at IALCH, in particular, the imaging findings, intervention and subsequent outcomes.

**Results:**

Computed tomography (CT) was the most utilised imaging modality prior to patients undergoing DSA. A dilated superior ophthalmic vein (96%) was the most prevalent imaging finding on axial imaging. At DSA, all except two patients had high-flow fistulas. The fistulas predominantly drained anteriorly (69.44%) and a cavernous internal carotid artery aneurysm was identified in eight patients. Occlusion of the fistula was attained in all patients that were compliant with follow-up and underwent intervention (*n* = 36, 100%), but parent artery sacrifice was required in 10 cases (27.78%).

**Conclusion:**

A wide range of imaging modalities can be used in the workup of a CCF. CT is currently the most accessible modality in our setting, with limited access to magnetic resonance imaging. On axial imaging, a dilated superior ophthalmic vein is the commonest finding. Classification of a fistula according to flow dynamics and noting the presence of aneurysms or pseudoaneurysms was found to be more practical in comparison to the traditional Barrow’s classification. Management outcomes at our institution compare well with available local and international data.

## Introduction

**FIGURE 1:**
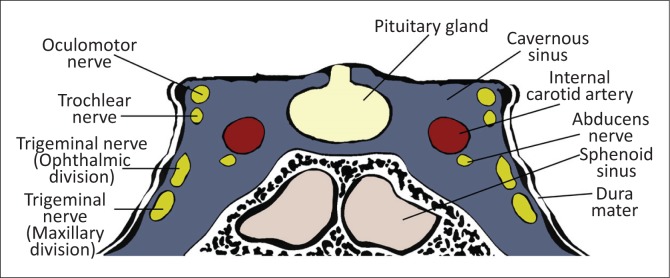
Schematic diagram depicting anatomical relationship between structures within the cavernous sinus.

**FIGURE 2:**
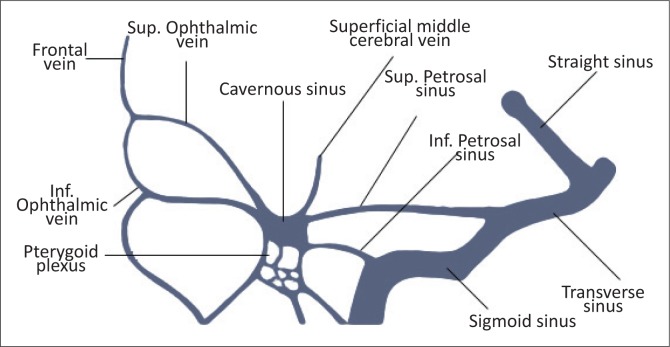
Tributaries and drainage of cavernous sinus.

The cavernous sinuses (CS) are paired dural venous sinuses that form part of a complex network of venous channels into which the superficial venous system of the head drains. It receives venous blood from the superior and inferior ophthalmic veins, Sylvian veins and pterygoid venous plexus and in turn empties into the superior and inferior petrosal sinuses which ultimately drain via the internal jugular vein. Because of its location in the parasellar region, many neurovascular structures (including the internal carotid artery [ICA]) traverse the sinus providing an opportunity for complex interactions to occur between the structures (see Figures 1 and 2).

The ICA has a tortuous path and is divided into segments. After passing through the superior part of the foramen lacerum to enter the cranial cavity, the ICA reaches the petroclinoid ligament where it passes anteriorly between the layers of the dura that form the CS taking up a medial position within the sinus. The ICA then exits the CS in the region of the anterior clinoid process. Branches of the ICA arising within the CS are the meningohypophyseal artery (MHA), inferolateral trunk (ILT) and capsular artery which in turn give off sub-branches supplying the dural covering of the CS. The medial wall of the CS is supplied by branches of the MHA and capsular arteries whilst the inferior and lateral walls are supplied by the ILT. The ICA’s companion branch, the external carotid artery (ECA), whilst supplying the major structures of the head and neck, also supplies parts of the meningeal layers in the cavernous region of the brain via distal sub-branches such as the middle meningeal artery which anastomose with distal branches of the ILT.

Carotid cavernous fistulas (CCFs) are abnormal arteriovenous communications between the carotid circulation and the CS, resulting in increased CS pressure and siphoning of the cerebral arterial supply. The CCF may be spontaneous or traumatic and have a variable flow dynamic (high vs. low flow).^1^

The Barrow classification is the most popular system used to classify CCFs. The classification categorises fistulas according to their arterial supply. Type A fistulas are direct communications between the cavernous segment of the ICA and the CS. Types B, C and D are indirect communications between the CS and dural meningeal branches of the ICA or ECA. Direct fistulas (Type A) are usually accompanied with high flow rates and are mostly a consequence of craniofacial fractures, aneurysmal rupture or surgical intervention. Rarely, diseases causing weakness of the arterial walls may predispose to the development of a spontaneous direct fistula. Indirect fistulas (Types B, C and D) have an unclear aetiology and appear to be more prevalent in post-menopausal, diabetic and hypertensive women. Other risk factors include collagenopathies, CS thrombosis, sinusitis and pregnancy. Indirect fistulas have low flow rates and are usually spontaneous.^2,3^

Carotid cavernous fistulas are infrequent. Direct fistulas are more common than the indirect types but just 0.2% of patients with traumatic brain injuries go on to develop direct fistulas.^4,5^

Carotid cavernous fistulas have a varied clinical presentation (ocular symptoms, cranial nerve neuropathies, headache or pulsatile tinnitus) which is dependent on the type, flow dynamics, size, location, route of venous drainage and duration of the fistula. Fistulas may complicate and result in intracranial haemorrhage and ischaemia. Smaller fistulas may remain asymptomatic.^1,2^

Multiple imaging modalities are used to make the diagnosis of a CCF. Digital subtraction angiography (DSA) is considered the gold standard to diagnose and plan therapy and intervene when necessary. Computed tomography (CT) and magnetic resonance imaging (MRI) are valuable and are quite often the initial imaging modalities used. Ultrasonography (US) is a more recent addition to the initial diagnostic workup.^4^

Digital subtraction angiography whilst being invasive is necessary for making an accurate anatomical diagnosis and for the planning of further management by identifying the exact location of the breach in the affected artery, the drainage pattern and the possible contributing factors such as thrombosis and aneurysms. At angiography, distal flow and cerebral collateral circulation is also assessed in the event that the ICA needs to be sacrificed in the course of management.^4,6^

Treatment options in CCFs are based on the type of fistula, degree of clinical symptoms and risk of complications. Treatment options in indirect fistulas with low risk of complications include conservative treatment and radiosurgery. High-flow, direct fistulas or failed conservative treatment of low flow fistulas requires endovascular or surgical intervention.^6^

At intervention, the aim of repair is to occlude the site of communication whilst ensuring the ICA remains patent. If the defect is too large or if embolisation fails, the parent artery (ICA) is sacrificed after assessing the adequacy of collateral cerebral blood flow.

## Research method and design

Formal ethics approval was obtained from the Biomedical Research Ethics Committee (BREC), College of Health Sciences, University of KwaZulu-Natal (BREC Ref No.: BE475/16). Data were collected retrospectively from all patients presenting with CCF that were managed by the Department of Neurosurgery at Inkosi Albert Luthuli Central Hospital (IALCH) in Durban, South Africa, from January 2003 to May 2016.

The imaging modalities used in the workup and management of these patients included CT and MRI. Most of the preliminary imaging was carried out at various base hospitals and referring centres prior to the patients’ arrival at IALCH. The available reports and images of these studies were reviewed and analysed.

All patients subsequently underwent DSA and intervention, if necessary, at IALCH. Interventional procedures were performed in the angiography suite in the Radiology Department by an experienced neurosurgeon and/or interventional radiologist. Angiography was initially performed using a Siemens Axiom Artis FA Floor mounted machine. Subsequently, from 2011 onwards, angiography was performed using a Siemens Artis Q Zee Biplane machine. All patients underwent local or general anaesthesia where appropriate. Vascular access was obtained via the femoral artery and intravenous Heparin was administered to prevent thrombosis. A balloon occlusion test was performed on all patients to assess collateral blood flow should parent artery sacrifice need to be considered. DSA images were reviewed and data collected.

All data with categorical variables were presented as frequencies and proportions, and continuous variables were summarised by means or medians, where appropriate. The Shapiro–Wilk test was used to test for normality. Fisher’s exact test was used to test whether there were significant associations between categorical variables of interest.

## Results

**FIGURE 3:**
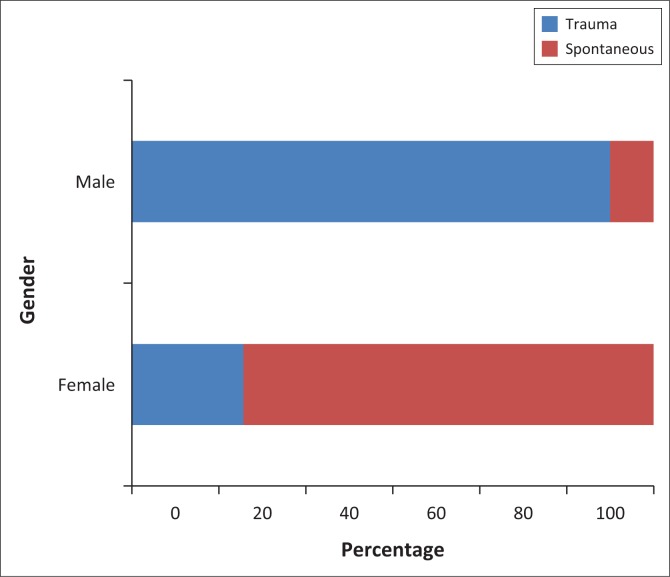
Relationship between gender and cause.

Initially, 38 patients were included in the study over the 13-year period. The mean age of the patients was 37.6 with a range of 9–94. The majority of patients were older than 30 years (60.52%). There were 24 male patients (63.16%) and 14 female patients (36.84%). In approximately two-thirds of the cases, the fistula was post-traumatic. A statistically significant difference was observed in the proportion of fistulas caused by trauma between men and women (91.67% vs. 21.4%, *p* < 0.001). This is presented in Figure 3.

**FIGURE 4:**
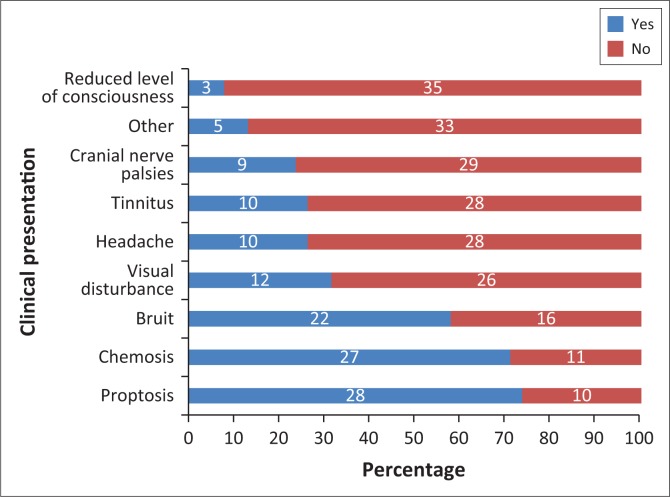
Clinical presentation observed in patients presenting with a carotid cavernous fistula.

Most patients presented with orbital and/or ocular signs (92.11%). Neurological signs and symptoms were noted in more than half of the patients (60.53%). The specific symptoms and signs are presented in Figure 4. The most prevalent sign was proptosis and the least prevalent was a depressed level of consciousness. Alteration of mental state in these patients was most likely as a consequence of other injuries sustained.

Patients were most frequently imaged with CT prior to angiography (81.58%, *n* = 31). There were five patients who underwent MRI only (13.16%) and three patients who had both a CT and an MRI (7.89%) prior to an angiogram. No prior imaging was performed in two patients who went straight to angiography. No documented usage of ultrasound in the initial workup was recorded.

Not all prior imaging reports or images were available for review as some patients had imaging at their referring hospital and either did not bring their imaging with them or the findings were not documented. A total of 13 patients had no available data prior to DSA regarding the findings on previous imaging or had no scan at all.

**FIGURE 5:**
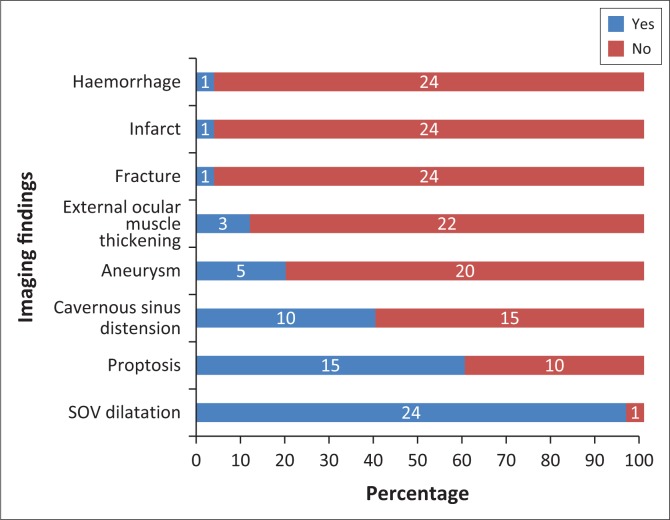
Imaging findings in patients presenting with a carotid cavernous fistula.

The imaging findings for the 25 patients with imaging data are presented in Figure 5. The most common finding was dilatation of the superior ophthalmic vein (96%) followed by proptosis (60%) and cavernous sinus distension (40%).

A total of four patients who did not undergo intervention or did not comply with follow-up were excluded from the subsequent analysis (*n* = 4). The reasons for exclusion are documented in Table 1. Although most patients presented with a single fistula, a few exceptions (*n* = 3) were noted with two simultaneous fistulas. For ease of statistical analysis, each fistula was analysed independently. A total of 36 fistulas in 34 patients were considered for analysis. DSA findings are presented in Table 2. The fistulas were classified according to their flow dynamic, cause and the presence of aneurysm or pseudoaneurysm. High-flow fistulas were present in all but two patients (*n* = 34, 94.44%). There was early filling of the affected CS in all patients. The fistulas did not favour a particular side and had predominantly anterior drainage only (*n* = 25, 69.44%). Aneurysms were identified in eight patients (22.22%) and psuedoaneurysms in two cases (5.56%). All patients passed the trial balloon occlusion test, suggesting adequate collateral blood flow.

**TABLE 1:** Patients excluded from digital subtraction angiography and outcome analysis.

Patient	Reason for exclusion
CASE 7	Coils initially used but with limited success. Patient subsequently defaulted follow-up.
CASE 21	Fistula noted at angiography to have spontaneously closed.
CASE 29	Patient had an intracranial bleed after a traumatic brain injury and was too ill to attempt intervention. Patient subsequently demised.
CASE 30	Three balloons used at initial angiography, resulting in markedly reduced flow; however, fistula still present. Patient subsequently defaulted follow-up.

**TABLE 2:** Digital subtraction angiography findings.

Variable	Category	*N*	%
Side of fistula	Right	17	47.22
	Left	19	52.78
Type	High flow:	34	94.44
	• With aneurysm	8	22.22
	• With pseudoaneurysm	2	5.56
	Low flow	2	5.56
Drainage	Anterior only	25	69.44
	Posterior only	4	11.11
	Anterior and posterior	7	19.44
Filling times	Early	36	100

**FIGURE 6:**
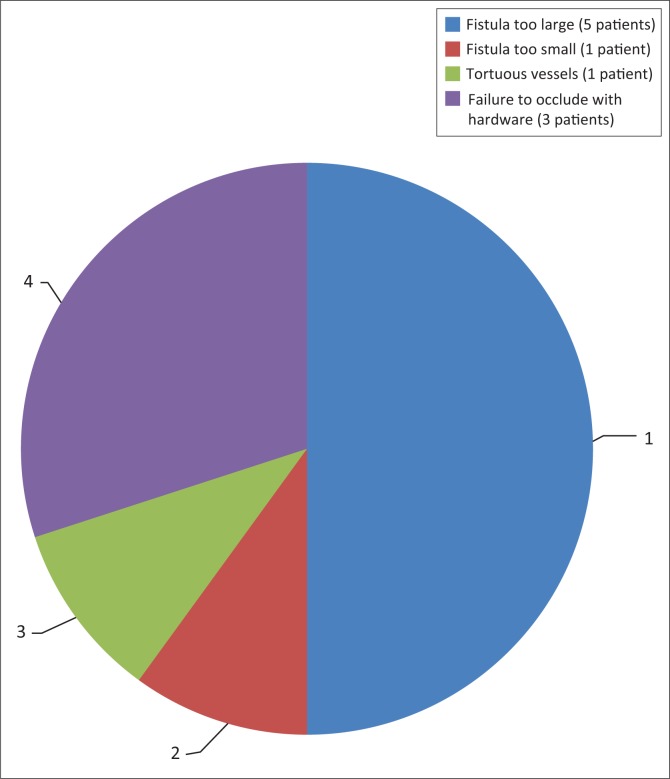
Reasons for internal carotid artery sacrifice in patients with carotid cavernous fistulas undergoing endovascular intervention (*n* = 10).

Fistula occlusion was attained via embolisation or trapping in all cases that were compliant with management and in whom intervention was attempted (*n* = 36, 100%). Sacrifice of the affected ICA was required in 10 patients (27.78%). Reasons for sacrifice are demonstrated in Figure 6. No documented post-procedural complications were noted.

One of the patients presented post-trauma, with bilateral high-flow fistulas. Multiple balloons were deployed bilaterally, which resulted in reduced filling but persistence of the fistula. A repeat angiogram was then performed at a later date. The right fistula, which was smaller, was treated successfully using a covered stent (Jo stent). The left fistula, however, was larger and required sacrifice of the left ICA to occlude the fistula. In Table 3, the relationship between outcome and demographic data, the side of fistula and presence of an aneurysm, respectively, is presented. Sacrifice of the ICA was slightly higher in those who had an intracavernous ICA aneurysm compared to those who did not (37.5% vs. 25%); however, this finding was not statistically significant (*p* = 0.658). A higher rate of ICA sacrifice was also observed in fistulas on the right side (35.3% vs. 21.1%); this result was also not statistically significant (*p* = 0.463).

**TABLE 3:** Relationship between outcome and various factors.

Variable	ICA not sacrificed (*N* = 26)				ICA sacrificed (*N* = 10)				*p*
	*N*	%	Mean	SD	*N*	%	Mean	SD	
**Age**	-	-	38.3	20.3	-	-	35.4	21.4	0.703
**Gender**	-	-	-	-	-	-	-	-	0.700
Male	18	75	-	-	6	25	-	-	
Female	8	66.7	-	-	4	33.3	-	-	
**Cause of fistula**	-	-	-	-	-	-	-	-	0.639
Spontaneous	8	66.7	-	-	4	33	-	-	
Trauma	18	75	-	-	6	25	-	-	
**Side of fistula**	-	-	-	-	-	-	-	-	0.463
Right	11	64.7	-	-	6	35.3	-	-	
Left	15	78.9	-	-	4	21.1	-	-	
**Aneurysm**	-	-	-	-	-	-	-	-	0.658
Absent	21	75	-	-	7	25	-	-	
Present	5	62.5	-	-	3	37.5	-	-	

ICA, internal carotid artery; SD, standard deviation.

## Discussion

With the advent of CT and MRI, diagnosis of CCFs via non-invasive means has become easier. Most patients at our centre underwent CT prior to angiography. However, access to MR imaging in a limited resource setting still remains a challenge as only eight patients in total had MRI as part of their diagnostic workup.

**FIGURE 7:**
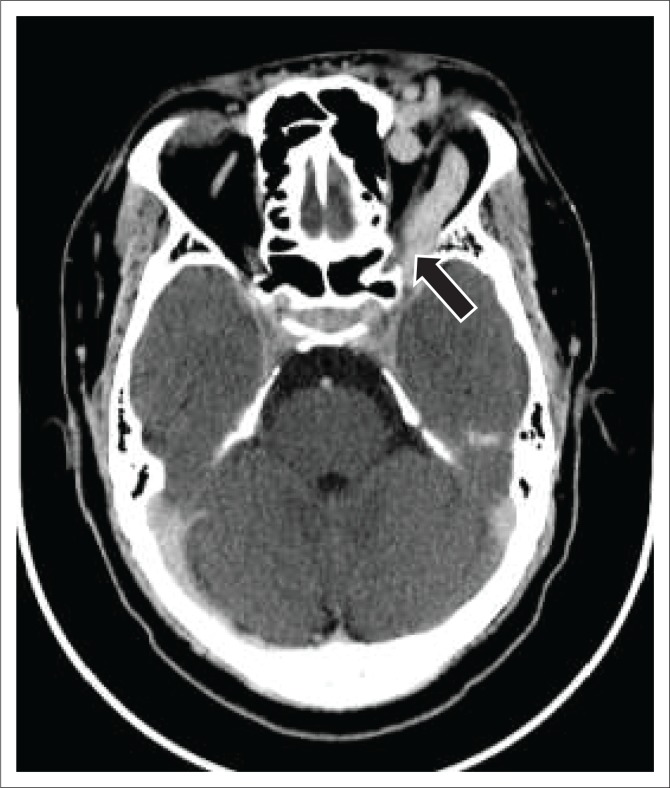
A 33-year-old male presented with a post-traumatic carotid cavernous fistula. Axial post-contrast computed tomography brain image demonstrates an enlarged tortuous left superior ophthalmic vein (arrow).

A dilated superior ophthalmic vein is often the initial finding on imaging and was found in 96% of the reviewed image data in our study, which compared favourably to other reports of 86% – 100% on enhanced CT and 75% – 100% on T1W or post-contrast MRI.^4^ Other findings on axial imaging include proptosis, retrobulbar fat stranding, sinus distension and early/avid enhancement of the affected CS (see Figures 7 and 9). In addition, CT allows easier identification of fractures and complications such as haemorrhage, whereas MRI allows the detection of abnormal flow voids within the CS. Magnetic resonance is understood to be superior to CT in detection of subtle signs.^4^

**FIGURE 8:**
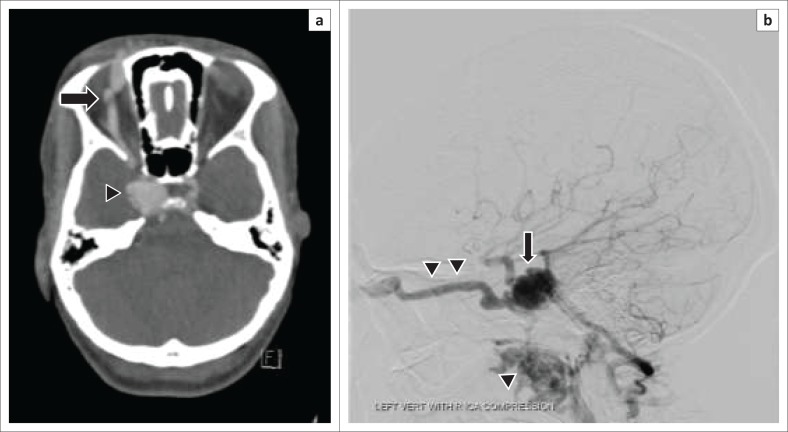
A 24-year-old female presented with a spontaneous history of proptosis, visual disturbance and pulsatile tinnitus: (a) Axial source image from a computed tomography angiography demonstrates early enhancement of a distended right cavernous sinus (arrowhead) and a dilated ipsilateral superior ophthalmic vein (arrow). (b) Lateral left vertebral artery angiogram with carotid compression (Heuber manoeuvre) in the same patient displays early filling of the distended cavernous sinus via the fistula (arrow) with drainage via anterior and inferior venous pathways (arrowheads).

**FIGURE 9:**
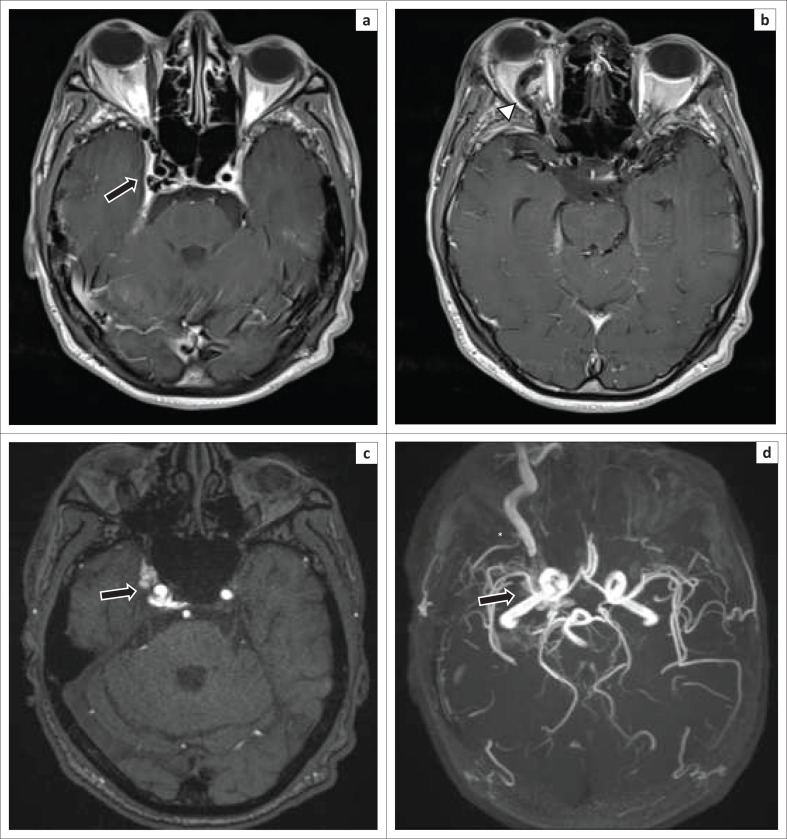
A 39-year-old male presented with proptosis and chemosis 6 months after being involved in a motor vehicle accident: (a) Axial T2W magnetic resonance imaging shows abnormal flow voids within the right cavernous sinus (arrow) as well as proptosis of the right globe. (b) Additional T2W slices in the same patient demonstrate a dilated ipsilateral superior ophthalmic vein (arrowhead). (c) Flow-related hyperintensity is noted on the MR time-of-flight (TOF) source images. (d) The corresponding MR TOF 3D MIP images display a dilated right superior ophthalmic vein (asterisk) with enhancement in the region of the right cavernous sinus (arrow).

Detection of flow-related hyperintensity within the affected CS on MR sequences such as three-dimensional Fast Imaging with Steady State Precession (FISP) and three-dimensional time-of-flight source images add value, especially in the event of subtle or absent findings on standard spin echo MR sequences.^7^

Non-invasive angiography techniques such as Computed tomography angiography (CTA) and MR angiography (MRA) sometimes allow determination of the size and exact location of the fistula (see Figure 8). The administration of Gadolinium does not provide any additional benefit.^7^

Transcranial US is well known to be cost-effective and non-invasive. CCF evaluation includes views through the temporal, orbital and carotid windows. The CS are identified as a symmetric cluster of flow on either side of the anterior clinoid process. Asymmetry of these structures suggests a possible fistula. On Doppler US, the superior orbital vein may show arterialisation, increased velocity and dilatation secondary to reversal of flow.^4^ Although most fistulas drain anteriorly to the ophthalmic veins, these orbital features may be absent in other patterns of drainage and may result in missed diagnosis. Carotid evaluation shows a reduced resistive index on the affected side. A decrease in velocity within the cerebral arteries on the ipsilateral side because of steal phenomenon may be present^8^. Despite its ability to strongly suggest a fistula, ultrasound falls short in determining the precise size and location of the fistula and does not add much value in clinical practice.

Notwithstanding the advances of the other imaging modalities in their ability to diagnose a CCF, DSA remains the gold standard.^4^ This is mainly because normal non-invasive imaging does not exclude the diagnosis of a CCF and also because of the ability of angiography to identify fistula angioarchitecture whilst allowing simultaneous planning and intervention.

**FIGURE 10:**
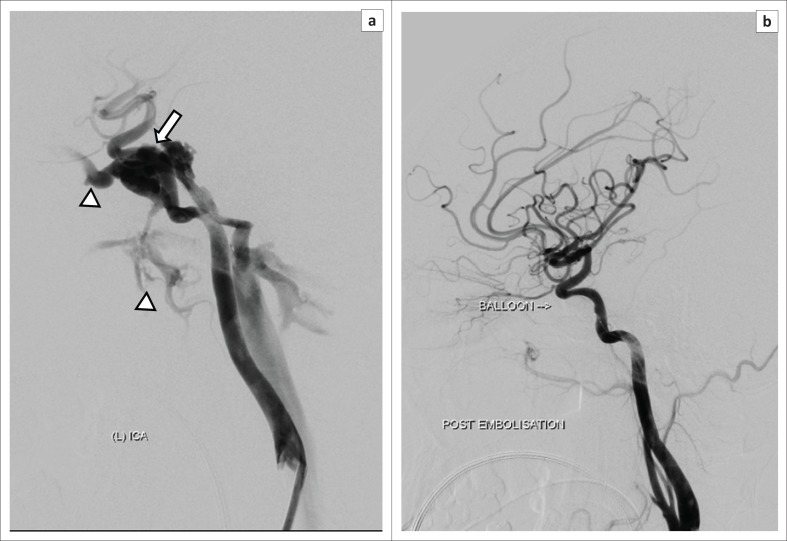
A 42-year-old female presented with proptosis, chemosis and an audible orbital bruit after sustaining a fall: (a) Selected digital subtraction angiography of the left internal carotid artery demonstrates early filling of the left cavernous sinus (arrow) with drainage via the anterior and inferior pathways (arrowhead). (b) Post-embolisation angiogram demonstrates successful occlusion of the fistula with preservation of the parent artery.

At DSA, the supplying arterial structures in low flow fistulas, the breech in the vessel wall in high-flow fistulas and venous drainage pattern can be easily assessed.^4^ Early contrast enhancement of the affected CS usually indicates the presence of a fistula (see Figure 10). Anterior venous drainage patterns are the commonest drainage pathway^1^ which is further supported by our study and accounts for the predominance of orbital/ocular signs. Specific assessment for cortical venous drainage at angiography is advised as urgent treatment is indicated, if present, because of its association with potentially life-threatening sub-arachnoid/intracerebral haemorrhage.^9^

A six-vessel angiogram is recommended over a four-vessel study as a small low flow fistula may be missed. If the fistula is not identified because of high flow, manoeuvres such as the Mehringer-Hieshima and Heuber manoeuvres can be utilised to slow down the flow and allow identification of the fistula.^6^

**FIGURE 11:**
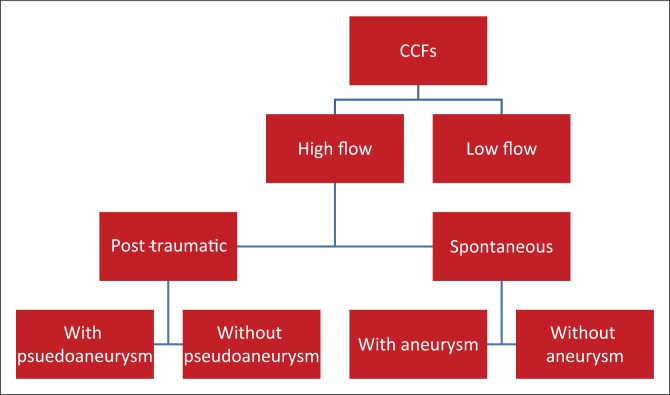
Classification system used during study.

Assessing the arterial supply allows classification of the CCF by means of the Barrow’s classification. This classification was found to be confusing and did not add further value in terms of further management. In our experience, classifying the fistula according to its flow dynamic and documenting the presence of an intracavernous aneurysm or pseudoaneurysm was found to be more practical (see Figure 11).

Ideal management involves occlusion of the fistula whilst ensuring the affected ICA remains patent. Conservative treatment is reserved for patients with indirect CCFs and who have tolerable and few symptoms. For all other fistulas requiring intervention, occlusion via endovascular means is the preferred method.^3^ To accomplish this, agents such as detachable balloons, coils, embolic materials and stents can be utilised via a transarterial or transvenous route.^10^ If the fistula cannot be occluded, sacrifice of the affected ICA may be required. Surgical treatment is reserved when the endovascular approach is unsuccessful. Emergent intervention is sometimes required if angiographic or clinically poor prognostic features are present (see Table 4).^9^

**TABLE 4:** Poor prognostic features necessitating emergent treatment.

Angiographic	Clinical
Psuedoaneurysm	Raised intracranial pressure
Large varix of the cavernous sinus	Progressive proptosis
Cortical venous drainage	Diminished visual acuity
Thrombosis of other outflow venous structures	Major external haemorrhage (e.g. otorrhagia or epistaxis)
-	Intracerebral or sub-arachnoid haemorrhage
-	Transient ischaemic attacks

It is preferable to preserve the parent artery, as patients may develop immediate or delayed neurological deficits as a result of ischaemia,^11^ although this was not our experience. Furthermore, the development of a new cerebral aneurysm or evolution of existing aneurysms and subsequent aneurysmal rupture as a result of altered cerebral haemodynamics in patients with parent artery sacrifice has not been convincingly disproved.^12^

Over the years, worldwide studies have showed varying degrees of success at achieving the ideal outcome. Internal carotid artery preservation rate in reviewed studies ranged between 40% and 84%^13,14^ and CCF occlusion between 86% and 100%.^13,15^ To date, scant local data exist to our knowledge. In a single local study, Szkup and Beningfield,^16^ in their series of 34 cases, documented a preserved parent artery in approximately 53% of their cases and attained occlusion in 91%. Most of the cases requiring sacrifice were because of large tears in the affected vessel generally secondary to trauma. The results at our centre, of occlusion in all (100%) of the cases and a 72.22% ICA preservation, is similar in comparison to the reviewed local and international data.

Spontaneous direct CCFs are commonly secondary to rupture of an intracavernous ICA aneurysm. In our study, 37.5% (*n* = 3) of patients with such an aneurysm ultimately required sacrifice of their ICA. Although this relationship was not statistically significant, studies suggest that parent artery occlusion is sometimes a necessary choice if first-line methods using detachable balloons or coils fail.^17^

### Limitations of the study

Not all prior imaging data were available for review, particularly imaging that was performed at referring institutions and hospitals. With recent and continued improvement being made in the way data are stored at our local facilities, perhaps a more comprehensive follow-up study can be performed at a later date.

## Conclusion

Initial imaging in our locale was dominated by CT whilst MR imaging was reserved for a few cases because of accessibility. US, which is thought to have little clinical value, was not utilised. A dilated superior ophthalmic vein is usually present on imaging, especially if the fistula is accompanied by anterior venous drainage. Caution should be taken in the absence of this sign to not miss a fistula with an alternate drainage pathway.

Digital subtraction angiography remains the gold standard allowing diagnostic confirmation, management planning and therapeutic intervention. Classification of the fistulas according to flow dynamic and noting the presence of aneurysms or pseudoaneurysms was found to be more practical in comparison to the traditional Barrow’s classification. The outcomes of 100% fistula occlusion and 72.22% ICA preservation compares well with available local and international data.
